# Femoral Valgus Correction Angle for the Intramedullary Alignment Rod Is Strongly Associated with Femoral Lateral Bowing in Japanese Patients with Varus Knee Osteoarthritis Undergoing Total Knee Arthroplasty

**DOI:** 10.1155/2022/7223534

**Published:** 2022-08-16

**Authors:** Yosuke Hattori, Nobuyuki Asai, Shotaro Mori, Ken Ikuta, Yusuke Kazama, Yusuke Iesaki, Shimpei Takahashi, Atsushi Kaneko, Tomotaro Sato

**Affiliations:** National Hospital Organization Nagoya Medical Center, Orthopaedic Surgery and Rheumatology, Nagoya, Japan

## Abstract

**Background:**

This study aimed to investigate factors, such as differences in femoral shape, that could affect the femoral valgus correction angle (VCA) for the intramedullary alignment rod (IM rod) by using a three-dimensional (3D) measurement system in patients with varus knee osteoarthritis undergoing total knee arthroplasty (TKA).

**Methods:**

A total of 305 knees in 233 Japanese patients with varus knee osteoarthritis who underwent primary TKA by using Jig Engaged 3D Pre-Operative Planning Software for the TKA operation support system was examined. We retrospectively analysed factors, such as the shape of the proximal, middle, and distal femur in the coronal plane, all of which could affect the VCA for the IM rod, by multiple linear regression analyses.

**Results:**

The VCA for the IM rod was 5.9° ± 1.6° (range: 1.7° to 10.7°), and the femoral lateral bowing angle (FBA) was 3.5° ± 3.2°. Major factors independently associated with the VCA for the IM rod were the FBA (*β*: 0.75), femoral offset (*β*: 0.38), and the medial angle between the mechanical femoral axis and the line that connects the distal margins of the medial and lateral femoral condyles (*β*: −0.16). The model was created by stepwise multiple linear regression (*F* = 266.6, *p* < 0.001, and estimated effect size = 4.4) explained 85% of the variance in the VCA for the IM rod (*R*^2^ = 0.85).

**Conclusions:**

The VCA for the IM rod was most strongly associated with femoral lateral bowing in patients with varus knee osteoarthritis undergoing TKA. Our findings suggest that preoperatively measuring the VCA for the IM rod in patients with femoral lateral bowing by using a 3D measurement system could be useful for accurate coronal alignment of the femoral component in TKA.

## 1. Introduction

Postoperative neutral limb alignment is an important factor for successful total knee arthroplasty (TKA) using the standard mechanical alignment method, as improper alignment leads to surgical revision due to wear, aseptic loosening, and instability [1–3]. Postoperative coronal alignment is believed to align the femoral and tibial components perpendicularly to the mechanical axes of the femur and tibia to achieve a neutral alignment [4, 5]. The alignment of the limb is primarily controlled by the distal femur as well as proximal tibial cuts, and most surgeons agree that postoperative limb alignment should be corrected within 0° ± 3° of the mechanical axis [6, 7].

It is a standard practice in TKA to align the intramedullary alignment rod (IM rod) with the anatomical femoral axis, i.e., a line drawn along the center of the intramedullary canal. The femoral valgus correction angle (VCA) for the IM rod is determined by the angle formed by the anatomical and mechanical femoral axes on preoperative planning. The use of preoperative planning to determine the VCA for the IM rod has been emphasized for nonnavigated TKA to ensure accurate distal femoral resection [8].

Few studies have examined relationships between the VCA for the IM rod and variations in the shape of the proximal, middle, and distal femur in the coronal plane in patients with osteoarthritis by using long-standing radiographs. In addition, it is difficult to evaluate angular parameters by using preoperative long-standing radiographs accurately. To this end, the present study aimed to investigate factors, such as differences in femoral shape, that could affect the VCA for the IM rod in patients with varus knee osteoarthritis undergoing TKA by using a 3D measurement system.

## 2. Materials and Methods

**Figure 1:**
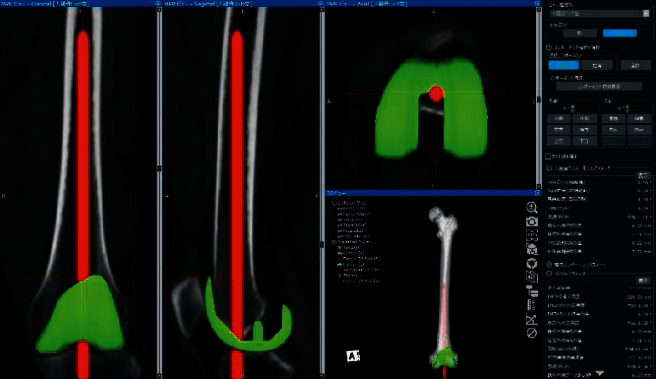
Preoperative planning using Jig Engaged 3D Pre-Operative Planning Software for the TKA operation support system.

A retrospective analysis of factors that could affect the VCA for the IM rod was conducted in Japanese patients who underwent primary TKA due to osteoarthritis from October 2014 to December 2019 at one institution. The study group consisted of 305 consecutive knees in 233 patients (44 men and 189 women) with varus knee osteoarthritis who underwent TKA with preoperative planning by using Jig Engaged 3D Pre-Operative Planning Software for the TKA (JIGEN®) operation support system (LEXI Inc., Tokyo, Japan) [9]. This program creates 3D models of the bone using computed tomography (CT) data and uses reference points and coordinate axes to calculate parameters regarding the positions of components (e.g., flexion, rotation, and resection amount), the IM rod, and the surgical jig (ArthroDesign Ltd., Kawaguchi, Japan). Selected implants can be placed at optimal positions on the 3D model and multiplanar reconstruction images. Femoral components were set perpendicular to the mechanical femoral axis in the coronal plane by using the standard mechanical alignment method. After determining the insertion position, the IM rod was inserted to the maximum until the rod tip was in contact with the front cortex of the femoral medullary cavity. The VCA for the IM rod and the insertion depth of the IM rod were automatically calculated (Figure 1). All patients included in this study were treated at our institution and had neither severe distal femoral/tibial bone loss nor extra-articular deformity of the femur/tibia due to previous trauma or surgery (e.g., hip arthroplasty or internal fixation of a femoral fracture). The mean age of participants was 74.8 ± 8.1 (range, 42–94) years. The Kellgren–Lawrence classification of osteoarthritis was grade III in 104 knees and IV in 201 knees.

**Figure 2:**
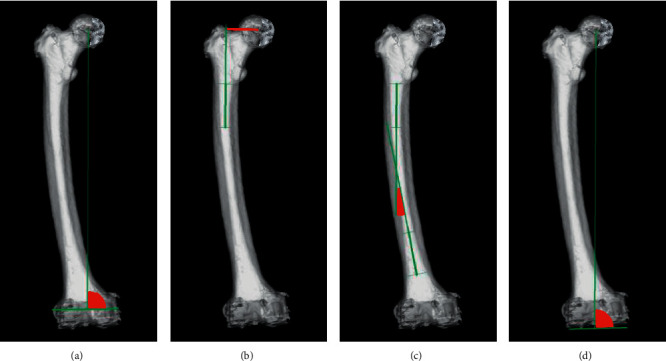
Shape of the proximal, middle, and distal femur in the coronal plane assessed by three-dimensional bone models. (a) MA-SEA angle, (b) femoral offset, (c) FBA, and (d) MA-DFC angle. MA–SEA angle: medial angle between the FMA and the SEA, FMA: femoral mechanical axis, SEA: surgical epicondylar axis, FBA: femoral lateral bowing angle, MA–DFC angle: medial angle between the FMA and the DFC, DFC: the line that connects the distal margins of the medial and lateral femoral condyles.

In addition to age, sex, and body mass index (BMI), the following variables were evaluated as potential factors that could affect the VCA for the IM rod: femoral offset, measured as the perpendicular distance from the center of the femoral head to the line bisecting the femur at 0 and 5 cm below the lowest portion of the lesser trochanter in the coronal plane (Figure 2(a)); femoral lateral bowing angle (FBA), measured as the angle between a line that bisects the femur at 0 and 5 cm below the lowest portion of the lesser trochanter and a line that connects the portions bisecting the femur at 5 and 10 cm above the distal articular surface in the coronal plane [10, 11] (Figure 2(b)); medial angle between the mechanical femoral axis and the line connecting the distal margins of the medial and lateral femoral condyles in the coronal plane (MA-DFC angle; Figure 2(c)); medial angle between the mechanical femoral axis and the surgical epicondylar axis (SEA) in the coronal plane (MA-SEA angle; Figure 2(d)). Lateral bowing was designated as positive (+) and medial bowing as negative (−).

All patients underwent a helical CT scan per protocol using low dose radiation (2–3 mSv). CT images were taken from the femoral head to the ankle in the axial slice direction with a slice pitch of 2 mm or less. The femoral coronal plane was defined as a coordinate system based on a plane consisting of the spherically approximated femoral head center and the SEA, i.e., the line connecting the prominence of the lateral epicondyle and the sulcus of the medial epicondyle. The mechanical femoral axis was defined as the line connecting the center of the femoral head and the portion bisecting the prominence of the lateral epicondyle and the sulcus of the medial epicondyle (the center of the knee). Parameters projected onto the femoral coronal plane, such as the VCA for the IM rod, MA-SEA angle, femoral offset, FBA, and MA-DFC angle, was calculated by this computer software which created the femoral coordinate system based on a femoral coronal plane. To evaluate intraobserver and interobserver reliability, 50 patients were randomly selected, and all angular parameters were measured twice by two observers, with a 4-week interval between measurements.

This study was approved by the ethics committee of our institution and was conducted in accordance with the Declaration of Helsinki. Written informed consent was obtained from all participants. Patient anonymity was maintained during data collection, and security of personal information was strictly controlled.

### 2.1. Statistical Analysis

All results are expressed as mean ± SD or percentage. Pearson's correlation coefficients were calculated to explore associations between variables. Stepwise multiple linear regression analysis was performed to examine if any of the following variables could affect the VCA for the IM rod: age, sex, BMI, femoral offset, the FBA, the MA-DFC angle, and the MA-SEA angle. Sample size analysis revealed that 293 knees would be needed for multiple linear regression analysis including seven predictors, a desired power of 0.80, an alpha-level of 0.05, and an estimated effect size (ES, Cohen's f2) of 0.05. To determine the incidence of apparent femoral bowing (defined as lateral or medial bowing >3°) and to evaluate the VCA for the IM rod according to the FBA, patients were divided into the following four groups: FBA < −3°, −3°≤ FBA ≤ 3°, 3°< FBA ≤ 6°, and 6°< FBA. This analysis was performed using the Kruskal–Wallis test. *p* < 0.05 was considered statistically significant. Intraobserver and interobserver reliabilities were determined with intraclass correlation coefficients (ICCs) for each measurement. Data were analysed using SPSS for Windows, version 22.0 (SPSS, Inc., Chicago, IL, USA).

## 3. Results

Patient demographics, clinical characteristics, and angular parameters are summarised in Table 1. The VCA for the IM rod was 5.9° ± 1.6° (range: 1.7° to 10.7°), and the FBA was 3.5° ± 3.2° (range: −4.5° to 14.2°). Correlations between assessed variables are shown in Table 2. The VCA for the IM rod was strongly correlated with the FBA (r: 0.79) and moderately correlated with femoral offset (r: 0.30), the MA-DFC angle (r: −0.51), and the MA-SEA angle (r: −0.34).  Table 3 shows the results of stepwise multiple linear regression analyses. The model created by stepwise multiple linear regression (*F* = 266.6, *p* < 0.001, ES = 4.4) explained 85% of the variance in the VCA for the IM rod (*R*^2^ = 0.85). The FBA (*β*: 0.75), femoral offset (*β*: 0.38), the MA-DFC angle (*β*: −0.16), female sex (*β*: 0.10), and the MA-SEA angle (*β*: −0.06) were independently associated with the VCA for the IM rod. Categorisation of the VCA for the IM rod according to the FBA is presented in Table 4. The group with an FBA > 6° had a higher mean VCA for the IM rod (7.9 ± 1.3) than the other three groups. ICCs of intraobserver reliability were >0.90, and ICCs of interobserver reliability were >0.85 for all angular measurements, suggesting high reproducibility. Accordingly, measurements obtained by one researcher were used for all subsequent analyses.

## 4. Discussion

The present study is the first to use a 3D measurement system to investigate factors, such as differences in the shape of the femur, that could affect the VCA for the IM rod (i.e., the angle between the anatomical and mechanical femoral axes) in patients with varus knee osteoarthritis who underwent TKA. The most important findings are as follows: [1] the mean VCA for the IM rod was 5.9°, and [2] the VCA for the IM rod was most strongly associated with femoral lateral bowing in Japanese patients with varus knee osteoarthritis undergoing TKA.

In previous studies, the angle formed by the anatomical and mechanical femoral axes was evaluated using preoperative long-standing anteroposterior radiographs. The angle between the anatomical and mechanical femoral axes is generally around 6° [11–20]. Several studies have reported wide variation in femoral shape and in the angle between the anatomical and mechanical femoral axes [4, 11, 13–20]. Thus, to ensure an accurate cut of the distal femur during TKA, the angle between the anatomical and mechanical femoral axes should be measured preoperatively. The present study used a 3D measurement system and obtained a mean VCA for the IM rod of 5.9° ± 1.6° (range, 1.7° to 10.7°), which is close to the standard distal femoral cut of 6°. Our findings also revealed that wide variations exist in the angle between the anatomical and mechanical femoral axes in Japanese patients with varus knee osteoarthritis.

Femoral lateral bowing is commonly observed in Asian patients with knee osteoarthritis [10, 21]. In conventional TKA, proper lower limb alignment in the coronal plane cannot be achieved in patients with femoral lateral bowing [20, 22–25]. Reportedly, individual VCA for distal femoral resection could enhance the accuracy of postoperative limb and femoral component alignment in the coronal plane [13, 20, 26–28]. Shi et al. [20] reported that the individual VCA for distal femoral resection achieves a better postoperative limb and femoral component alignment than the fixed VCA technique for TKA in patients with femoral lateral bowing. Palanisami et al. [19] reported that individualization of VCA, which is highly variable and influenced by femoral lateral bowing, the femoral neck-shaft angle, and preoperative deformity, is preferable in patients with moderate to severe varus deformity. In the present study, we found an important interindividual variability in the VCA for the IM rod by using an accurate 3D measurement system. For clinical relevance, the individual VCA for distal femoral resection can be more reliable and reproducible for the accuracy of postoperative limb and femoral component alignment in TKA. Kim et al. [11] reported that apparent femoral lateral bowing (i.e., FBA > 3°) was observed in 37 (11.7%) of 316 consecutive Korean patients with osteoarthritis who underwent primary TKA using preoperative long-standing anteroposterior radiographs. They also reported that the angle between the anatomical and mechanical femoral axes was mainly influenced by femoral shaft bowing in the coronal plane among patients with femoral deformities. In the present study as well, the VCA for the IM rod was found to be strongly associated with femoral lateral bowing in the coronal plane. In addition, the influence of femoral lateral bowing on VCA for the IM rod was stronger than that of femoral offset in patients with varus knee osteoarthritis undergoing TKA. We observed femoral lateral bowing (i.e., FBA > 3°) in 152 (49.8%) of 305 knees; notably, apparent femoral lateral bowing (i.e., FBA > 6°) was observed in 63 (20.7%), with the maximum FBA being 13.0°. Thus, the distal femoral cut with a fixed 6° valgus angle may result in postoperative limb malalignment in patients with apparent femoral lateral bowing. We also found a strong positive correlation between the VCA for the IM rod and the FBA. On the other hand, the VCA for the IM rod was weakly associated with the MA-DFC angle and the MA-SEA angle, which represent the shape of the distal femur. These findings suggest that the amount of distal bone resection of the lateral femoral condyle does not increase abnormally as the VCA for the IM rod increases in patients with apparent femoral lateral bowing.

The need to evaluate femoral lateral bowing and measure the VCA for the IM rod in preoperative long-standing anteroposterior radiographs has been suggested. This is based on the premise that long-standing radiographs covering the entire femur are taken in the frontal position of the exact femur. However, a variation in patient position during acquisition of long-standing radiographs may result in errors of up to 2° when calculating the leg axis [29, 30]. In long-standing radiographs of the knee deformed in varus due to osteoarthritis, femoral lateral bowing could be overestimated by the influence of original femoral bowing due to the external rotational position of the hip joint. In the present study, preoperative planning with a coordinate system for the central axis of the IM rod which was constructed in 3D with a new operation support system was able to eliminate errors resulting from uncorrected leg position in long-standing radiographs. The orientation of the IM rod, when fully inserted into the femoral medullary cavity, can be accurately defined as the anatomical femoral axis in preoperative planning by using the new operation support system, although it is difficult to measure the anatomical femoral axis accurately in patients with apparent femoral lateral bowing. The present study is the first to examine angular parameters, including the angle between the anatomical and mechanical femoral axes, using a 3D measurement system. Our findings suggest the usefulness of preoperative measurement of the VCA for the IM rod in achieving accurate coronal alignment of the femoral component in TKA in patients with variations in shape of the middle femur with the 3D measurement system.

This study has some limitations. First, most patients were classified into Kellgren–Lawrence grade III or IV. Thus, disease progression may have impacted the measurements. Second, as there was no healthy control group, it remains unclear to what extent variations in femoral shape were due to osteoarthritis. Finally, all samples were obtained from Japanese patients. Thus, our results may not be fully applicable to other ethnicities.

MA-SEA angle: medial angle between the FMA and the SEA, FMA: femoral mechanical axis, SEA: surgical epicondylar axis, FBA: femoral lateral bowing angle, MA-DFC angle: medial angle between the FMA and the DFC, DFC: the line that connects the distal margins of the medial and lateral femoral condyles.

## 5. Conclusion

We investigated factors, such as differences in the proximal, middle, and distal femur, that could affect the VCA for the IM rod by using a 3D measurement system in patients with varus knee osteoarthritis undergoing TKA. The mean VCA for the IM rod was 5.9°, and the VCA for the IM rod was most strongly associated with femoral lateral bowing in this patient population. Our findings suggest that preoperative measurements of the VCA for the IM rod in patients with femoral lateral bowing using a 3D measurement system could be useful for accurate coronal alignment of the femoral component in TKA.

## Figures and Tables

**Table 1:** Patient demographics, clinical characteristics, and angular parameters.

Age (years)	74.0 ± 8.1	(42, 94)
Female (%)	81.1	
BMI (kg/m^2^)	25.8 ± 4.3	(15.7, 41.1)
FTA (^o^)	183.4 ± 4.6	(167.8, 195.1)
Femoral offset (mm)	34.7 ± 4.4	(14.6, 46.0)
FBA	3.5 ± 3.2	(−4.5, 14.2)
MA-DFC angle (^o^)	90.8 ± 2.6	(81.3, 98.3)
MA-SEA angle (^o^)	90.6 ± 2.4	(84.4, 99.1)
VCA for the IM rod	5.9 ± 1.6	(1.7, 10.7)

Values are presented as the mean ± standard deviation (range). BMI: body mass index, FTA: femorotibial angle, FBA: femoral lateral bowing angle, MA-DFC angle: medial angle between the FMA and the DFC, FMA: femoral mechanical axis, DFC: the line that connects the distal margins of the medial and lateral femoral condyles, MA-SEA angle: medial angle between the FMA and the SEA, SEA: surgical epicondylar axis, VCA for the IM rod: femoral valgus correction angle for the intramedullary alignment rod.

**Table 2:** Correlations between assessed variables.

		VCA for the IM rod	Age	Female	BMI	Femoral offset	FBA	MA-DFC angle	MA-SEA angle
VCA for the IM rod									
r		1.00							
*p*									

Age									
r		0.25	1.00						
*p*		<0.01							

Female									
r		0.17	−0.04	1.00					
*p*		<0.01	0.26						

BMI									
r		−0.15	−0.39	0.00	1.00				
*p*		<0.01	<0.01	0.48					

Femoral offset									
r		0.30	−0.03	−0.21	−0.10	1.00			
*p*		<0.01	0.33	<0.01	<0.05				

FBA									
r		0.79	0.27	0.19	−0.12	−0.13	1.00		
*p*		<0.01	<0.01	<0.01	<0.05	<0.05			

MA-DFC angle									
r		−0.51	−0.10	0.02	0.10	−0.16	−0.33	1.00	
*p*		<0.01	<0.05	0.362	<0.05	<0.01	<0.01		

MA-SEA angle									
r		−0.34	−0.07	−0.01	0.03	−0.13	−0.24	0.41	1.00
*p*		<0.01	0.101	0.41	0.287	<0.01	<0.01	<0.01	

r: correlation coefficient, VCA for the IM rod: femoral valgus correction angle for the intramedullary alignment rod, BMI: body mass index, FBA: femoral lateral bowing angle, MA-DFC angle: medial angle between the FMA and the DFC, FMA: femoral mechanical axis, DFC: the line that connects the distal margins of the medial and lateral femoral condyles, MA-SEA angle: medial angle between the FMA and the SEA, SEA: surgical epicondylar axis.

**Table 3:** Results of linear regression analysis on variables associated with the VCA for the IM rod.

Variable	Standardized *β*coefficient	95% CI	*p* value
FBA	0.75	(0.35, 0.40)	<0.001
Femoral offset	0.38	(0.12, 0.16)	<0.001
MA-DFC angle	−0.16	(−0.13, −0.06)	<0.001
Female	0.10	(0.22, 0.64)	<0.001
MA-SEA angle	−0.06	(−0.07, −0.002)	<0.05

*R* ^2^ = 0.85, ANOVA *p* < 0.001 VCA for the IM rod: femoral valgus correction angle for the intramedullary alignment rod, FBA: femoral lateral bowing angle, MA-DFC angle: medial angle between the FMA and the DFC, FMA: femoral mechanical axis, DFC: the line that connects the distal margins of the medial and lateral femoral condyles, MA-SEA angle: medial angle between the FMA and the SEA, SEA: surgical epicondylar axis, CI: confidence interval.

**Table 4:** Categorisation of the VCA for the IM rod according to the FBA.

FBA (^o^)	*n* (%)	VCA for the IM rod (^o^)	*p* value
FBA ≤ 3	4 (1.3)	3.3 ± 1.3	<0.001
−3 ≤ FBA ≤ 3	149 (48.9)	5.0 ± 1.0	
3 < FBA ≤ 6	89 (29.2)	6.1 ± 1.1	
6 < FBA	63 (20.7)	7.9 ± 1.3	

VCA for the IM rod: femoral valgus correction angle for the intramedullary alignment rod, FBA: femoral lateral bowing angle.

## Data Availability

The data used to support the findings of this study are available from the corresponding author upon request.
